# Diversity across Seasons of Culturable *Pseudomonas* from a Desiccation Lagoon in Cuatro Cienegas, Mexico

**DOI:** 10.1155/2012/201389

**Published:** 2012-10-09

**Authors:** Alejandra Rodríguez-Verdugo, Valeria Souza, Luis E. Eguiarte, Ana E. Escalante

**Affiliations:** ^1^Departamento de Ecología Evolutiva, Instituto de Ecología, Universidad Nacional Autónoma de México, Apartado Postal 70-275, 04510 México, DF, Mexico; ^2^Department of Ecology and Evolutionary Biology, University of California, Irvine, CA 92091, USA; ^3^Departamento de Ecología de la Biodiversidad, Instituto de Ecología, Universidad Nacional Autónoma de México, Apartado Postal 70-275, 04510 México, DF, Mexico

## Abstract

Cuatro Cienegas basin (CCB) is a biodiversity reservoir within the Chihuahuan desert that includes several water systems subject to marked seasonality. While several studies have focused on biodiversity inventories, this is the first study that describes seasonal changes in diversity within the basin. We sampled *Pseudomonas* populations from a seasonally variable water system at four different sampling dates (August 2003, January 2004, January 2005, and August 2005). A total of 70 *Pseudomonas* isolates across seasons were obtained, genotyped by fingerprinting (BOX-PCR), and taxonomically characterized by 16S rDNA sequencing. We found 35 unique genotypes, and two numerically dominant lineages (16S rDNA sequences) that made up 64% of the sample: *P. cuatrocienegasensis* and *P. otitidis*. We did not recover genotypes across seasons, but lineages reoccurred across seasons; *P. cuatrocienegasensis* was isolated exclusively in winter, while *P. otitidis* was only recovered in summer. We statistically show that taxonomic identity of isolates is not independent of the sampling season, and that winter and summer populations are different. In addition to the genetic description of populations, we show exploratory measures of growth rates at different temperatures, suggesting physiological differences between populations. Altogether, the results indicate seasonal changes in diversity of free-living aquatic *Pseudomonas* populations from CCB.

## 1. Introduction

The Cuatro Cienegas basin (CCB), in Mexico, has been described as an important biodiversity reservoir within the Chihuahuan desert. The basin consists of a small (<840 km^2^) intermontane valley that contains different water systems. Most of the aquatic habitats are ephemeral, not permanent or subject to marked seasonal fluctuations [1]. Moreover, most aquatic systems in the area are extremely oligotrophic due to the almost negligible phosphorous levels [2]. Despite this, CCB is one of only two North American desert ecosystems characterized by high levels of species endemism including vertebrates, invertebrates [1, 3], and more recently a considerable list of microbes either bentonic, planktonic, or part of stromatolites and microbial mats [4–7]. Using culture-independent approaches, gammaproteobacteria in CCB appears as a dominant group in the aquatic environments [4, 8]. Within proteobacteria, *Pseudomonas* is itself a dominant group, with ample distribution and new endemic lineages or species described within the basin [5, 9], as well as a clear dominance in some microbial mats [7]. The unusual levels of biodiversity and endemism have led to describe CCB as well as either a time machine [10] or a “microbial Galapagos” [1, 4, 11] and have made it priority for conservation efforts by (comisión nacional para el conocimiento y uso de la biodiversidad) CONABIO, the world wildlife fund (wwf), the ramsar convention on wetlands and man, and the biosphere (MAB)/UNESCO.

Previous studies in CCB have sought to describe the unusual levels of microbial diversity across environmental or geographic gradients [6, 12, 13], as well as to understand the evolutionary and ecological origins of the observed diversity [4, 10, 14, 15]. However, nothing has been done to characterize bacterial diversity across seasons, despite (1) the existence of analytical models that indicate that seasonal fluctuation can influence the origin and maintenance of diversity [16] and (2) the marked seasonality in many of the aquatic systems in the basin [1, 17].

To evaluate the changes in microbial diversity associated with seasonality in water systems of CCB, we characterize, for the first time, microbial diversity across seasons in one of the seasonally variable freshwater systems (desiccation lagoon). The studied system is relatively small, and evidence exists of the strong influence that its seasonal environmental changes have in genetic variation of fish [17]. Within this context, we analyzed a fraction of total microbial diversity, the culturable *Pseudomonas* populations, and hypothesize that seasonal variation of microbial populations should track seasonal changes of the desiccation lagoon.

We statistically show that taxonomic identity of isolates is not independent of the sampling season, and that winter and summer populations are different. In addition to the genetic description of populations, we show exploratory measures of growth rates at different temperatures, suggesting physiological differences between populations. Altogether, the results indicate seasonal changes in diversity of free-living aquatic *Pseudomonas* populations from CCB.

## 2. Materials and Methods

### 2.1. Study Site

**Figure 1:**
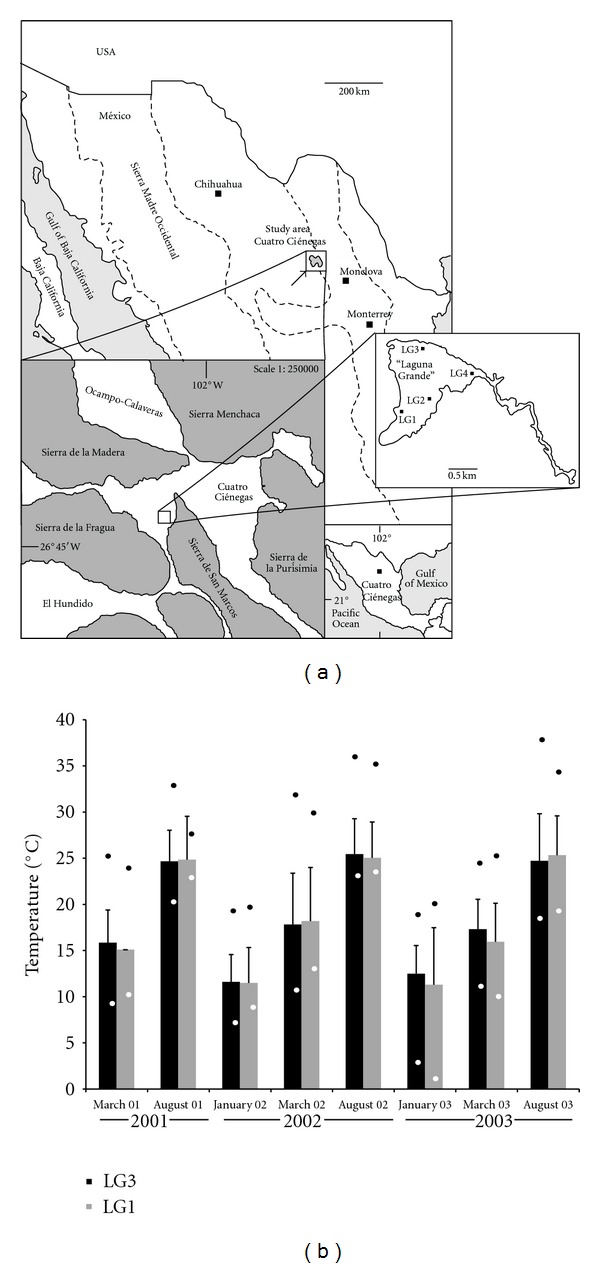
Study site in the Cuatro Cienegas basin. (a) Geographic location of the study site, indicating the sampling sited within “Laguna Grande” (desiccation lagoon) of the Churince system (modified from [8, 11]). (b) Average water temperature in two sites (LG1 and LG3) of “Laguna Grande.” Temperature was measured hourly over two weeks; average temperatures are significantly higher during summer than winter [17]. Error bars represent standard deviations, white dots and dark dots represent minimum and maximum temperatures, respectively [16].

We chose a seasonal aquatic ecosystem within CCB subject to marked fluctuations of chemical and physical parameters across seasons [18] temperature being one of them, as shown in Figure 1(b) (0–38°C range; [17]). The site is locally known as “Laguna Grande” (LG), and is located in the hydrological system of Churince on the western side of CCB (Figure 1(a)). Temperature was measured hourly over approximately two-week intervals at two sites (LG1 and LG3) using iButton temperature sensors (Maxim Integrated, Dallas, TX, USA).

### 2.2. Sampling and Isolation of Bacterial Strains

We sampled four sites in the desiccation lagoon “Laguna Grande”: LG1 (26°50.830′N, 102°09.335′W), LG2 (26°51.199′N, 102°09.009′W), LG3 (26°51.146′N, 102°08.964′W), and LG4 (26°51.222′N, 102°09.040′W). At a single time point, there were not significant temperature differences between sampling sites (Figure 1 and [6]), the multiple site sampling per time point was done to cover as much area as possible. Temperature variation was mostly through seasons, with temperatures reaching lows close to 0°C and highs close to 40°C (Figure 1; [17]). Samples were taken in summer (August 2003 and 2005) and winter (January 2004 and 2005). No further sampling was possible since 2006 because overexploitation of CCB aquifer associated with agricultural practices dried out the aquatic environment of “Laguna Grande.”

Triplicate samples of 15 mL of water were taken from surface water (15–20 cm depth) at each of the four samples sites using sterile BD Falcon vials (BD Biosciences, MA, USA). Each replicate sample was plated in triplicate by spreading 200 *μ*L of each vial. Culture plates contained GSP culture media (*Pseudomonas*-*Aeromonas* selective agar base): 10.0 (g L^−1^) sodium L(+) glutamate, 20.0 (g L^−1^) soluble starch, 2.0 (g L^−1^) potassium dihydrogen phosphate, 0.5 (g L^−1^) magnesium sulfate, 0.36 (g L^−1^) phenol red, and 12.0 (g L^−1^) agar-agar [19]. Strains that belong to the genus *Aeromonas* degrade the starch and produce acid, causing change in color (red to yellow). Strains that belong to the genus *Pseudomonas* did not produce acid; therefore, we selected the colonies that did not decolorize the media into yellow. The plates were incubated according to instructions for enrichments of *Pseudomonas*-*Aeromonas* [19]. Colonies were purified by subculturing on the same medium and maintained at −80°C in GSP media and 15% (w/v) glycerol.

### 2.3. DNA Extraction and BOX-PCR Genomic Fingerprint Analysis

DNA was extracted by using DNeasy Blood and Tissue Kit (Qiagen, CA, USA) according to the manufacturer's instructions. Repetitive extragenic palindromic PCR (rep-PCR) genomic fingerprinting of the isolates was carried out with a BOX-A1R primer (5′-CTACGGCAAGGCGACGCTGACG-3′) according to the protocol of [20]. The following PCR conditions were used: 7 min at 95°C, followed by 30 cycles of 94°C for 1 min, 53°C for 1 min, 65°C for 8 min, and a final extension at 65°C for 8 min. PCR products were analyzed on 1.5% (w/v) agarose gels containing 0.5X TAE-buffer (200 mM trisacetate, 0.5 mM EDTA, pH 8). The electrophoresis was performed for 5 hours at 180 mV (5 V cm^−1^). A 1-kb Plus DNA size ladder (INVITROGEN) was run at both sides and in the central lane of each gel. The gels were stained with ethidium bromide.

### 2.4. Computer-Assisted Analysis of BOX-PCR Genomic Fingerprints

Gel images were digitized with a charge-couple device video camera (Gel Logic 100, Kodak) and stored on disk as TIFF files. These digitized images were converted, normalized with the abovementioned DNA size markers, and analyzed with GelCompar software (version 4.0; Applied Maths, Kortrijk, Belgium). The “rolling disk” background subtraction method was applied. To analyse BOX-PCR patterns, similarity matrices of whole densitometric curves of the gel tracks were calculated by using the pair-wise Pearson's product-moment correlation coefficient (*r* value of 1 is equivalent to 100% similarity). This approach compares the whole densitometric curves of the fingerprints [21, 22]. Cluster analyses of similarity matrices were performed by the unweighted pair group method using arithmetic averages (UPGMA). We performed a cluster analysis of all DNA ladders to choose a similarity value to define isolates belonging to a same group of genotypes.

### 2.5. 16S rRNA Gene Sequencing and Analysis

We chose one isolate per genotype (as defined by rep-PCR analysis and determined by having at least 90% similarity in banding patterns) to obtain the 16S rDNA sequence. Previous studies have shown that clones with very similar BOX-PCR fingerprints (*r* values of more than 0.8) had identical 16S rRNA gene sequences [23]. The 16S rRNA gene was amplified using the 27F and 1492R primers under conditions described previously [24] in 100 *μ*L final volume. The PCR products were purified using the QIAquick gel extraction kit (Qiagen, Hilden, Germany). For sequencing the 16S rRNA gene (ca. 1450 bp) primers 27F, 357R, 530R, 530F, 790F, 981R, and 1492R were used [25]. The sequencing reaction had a total volume of 15 *μ*L consisting of 2 *μ*L Big Dye Terminator sequencing buffer (Applied Biosystems, Foster City, CA, USA), 1.6 *μ*M primer, and 5 *μ*L-purified amplified product. The amplification conditions were as follows: one cycle of 5 min at 95°C, and 45 cycles of 10 s at 95°C, 10 s at 50°C and 4 min at 60°C. Sequencing was done in a capillary sequencer (ABI-Avant 100). Sequences were assembled and revised using Consed software [26].

### 2.6. Nucleotide Accession Numbers

The 16S rRNA gene sequences obtained have been submitted to the GenBank database under accession numbers EU791282 and FJ976048-FJ976083.

### 2.7. Phylogenetic Analysis

The BLAST 2.0.6 algorithm of GenBank and the SIMILARITY_RANK tool of the Ribosomal Database Project II (RDP-II) were employed to search for closest matches found in the RDP-II and GenBank. Sequences were aligned using the CLUSTAL_W program [27]. Model generator (version 0.84, [28]) was used to determine the optimal nucleotide substitution model. Neighbor-joining (NJ) algorithm was used to generate a genealogy as implemented in PAUP (version 4.0, [29]), by using the GTR evolutionary model with gamma correction 0.40 and 1500 bootstrap replicates for all sequences.

### 2.8. Growth Rates

As an exploratory approach towards potential differences in physiological responses of winter and summer populations, growth curves at different temperatures were constructed, and maximum growth rates determined for a subset of isolates. The subset of isolates from the total sample represented winter and summer populations. The criteria for assembling this subset looked for a fair representation of genotype diversity at the individual level, as well as the inclusion of isolates that were obtained at different sampling dates and belong to the observed dominant lineages (*P. otitidis* and *P. cuatrocienegasensis*). By applying these criteria, the subset resulted in 6 genotypes of winter samples (*P. cuatrocienegasensis*) and 11 genotypes of summer samples (*P. otitidis*). We determined individual maximum growth rates at 5 different temperatures (28, 32, 26, 40, and 44°C), likely experienced in summer time, and ran the experiments in triplicate. A Biotek Synergy Microplate Reader (Synergy 2 Multi-Mode Microplate Reader Model, BioTek) was used to measure optical density of individual cultures every 10 min. Optical density measures were then used to construct growth curves and determine maximum growth rates.

### 2.9. Statistical Analyses

#### 2.9.1. Diversity Calculations and Genotypes

Genotypic diversity was obtained from the BOX-PCR fingerprinting. We calculated the index *G*/*N*, where *G* is the number of isolates with the same BOX-banding patterns and *N* the total number of isolates. The Shannon index of diversity was calculated using the formula: *H* = − ∑(*G*/*N*)ln⁡(*G*/*N*) [30]. The abundance of each genotype was calculated as the number of isolates in each genotypic group divided by total number of isolates. To determine how sampling effort affected these estimates, rarefaction curves were constructed comparing the number of isolates versus number of observed genotypes using ECOSIM (version 7.72) [31].

#### 2.9.2. Diversity Calculations and Phylotypes

Given the small sample size, and in order to evaluate diversity differences between summer and winter populations correcting for this, we constructed rarefaction curves [32] for the abundance of phylotypes (lineages) using ECOSIM (version 7.72) [31]. We also estimated the actual number of lineages (phylotypes) that may be present in the sample, by the calculation of a nonparametric Chao1 richness estimator using estimates 8.2.0 [33, 34].

To statistically determine the existence of two populations (summer and winter), we constructed a contingency table with the frequencies of lineages for the different sampling seasons and used a *G* test to evaluate the significance of our frequency distribution of lineages [35]. Finally, we performed a generalization of Fisher's exact test as using the Fisher test routine as provided in the *R* statistical package, using the simulate *P* value = TRUE flag.

#### 2.9.3. Comparisons of Growth Rates

Differences in growth rates at different temperatures were observed between summer and winter populations (*P. cuatrocienegasensis* and *P. otitidis*, resp.). To evaluate the statistical significance of these differences we performed a one-way analysis of variance as implemented in the *R* statistical package, using the function one-way test.

## 3. Results

To characterize the diversity of natural *Pseudomonas* isolates and its changes associated with seasonality in a CCB water system, we sampled a desiccation lagoon subject to marked seasonal fluctuations. Cultures were obtained from surface water samples in four sampling events (two summers, two winters). Individual isolates (70) were genotyped and temporal structure of the total sample analyzed.

### 3.1. Genetic Structure of Populations (Genotypic Diversity)

**Figure 2:**
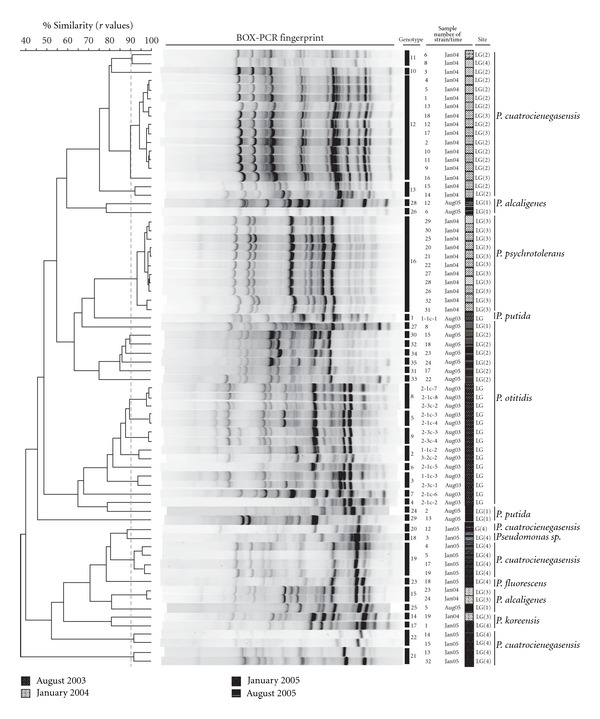
*Pseudomonas* isolates cluster analysis of genetic similarity. BOX-PCR genomic fingerprints of individual isolates were analyzed and grouped using product-moment UPGMA algorithm. A similarity value (*r*) of 90% was used to determine the same genotypes (dashed line).

Genotypic diversity was measured through genomic fingerprinting for each isolate using BOX-PCR technique, which permits the identification of individual clones, and each unique pattern was considered a different genotype. We chose a similarity value of 90% or more to indicate strains of the same (or very similar) genotype. Very similar or identical banding patterns have been demonstrated to have the same genotype and identical 16S rRNA gene sequences [23]. Cluster analysis resulted in a total of 35 representative genotypes (Figure 2). We identified 9 genotypes (15 isolates) from August 2003, 7 genotypes (31 isolates) from January 2004, 7 genotypes (12 isolates) from January 2005, and 12 genotypes (12 isolates) from August 2005.

The genotypic diversity calculated for the total sample (70 *Pseudomonas* isolates) and all estimates derived from this sample should be taken with caution given the small sample size. It has been said that the standard diversity description of the sample indicates that Shannon index (H) is 3.14. Additional analyses include the observation that genotypic diversity was heterogeneously distributed in the different samples. In January 2004, we observed the lowest diversity (*G*/*N* = 0.22) and the highest number of isolates having the same genotypic pattern (12 strains having the same genotype). While, in August 2005, we observed the highest diversity with 12 isolates out of 12 unique genotypes (*G*/*N* = 1). All genotypes were found to be unique to one sample occasion (Figure 2). Even when we applied a cutoff value of 80% to define clusters, the majority of genotypes (92.9%) were collected only once, except for two genotypes that included isolates from different sampling occasions. Rarefaction analysis showed that more sampling is needed to gain confidence on the genotype diversity present (data not shown). Thus, these observations are only suggestive of not reoccurrence of genotypes from year to year.

### 3.2. Seasonal Changes (*P. otitidis* and *P. cuatrocienegasensis*)

**Figure 3:**
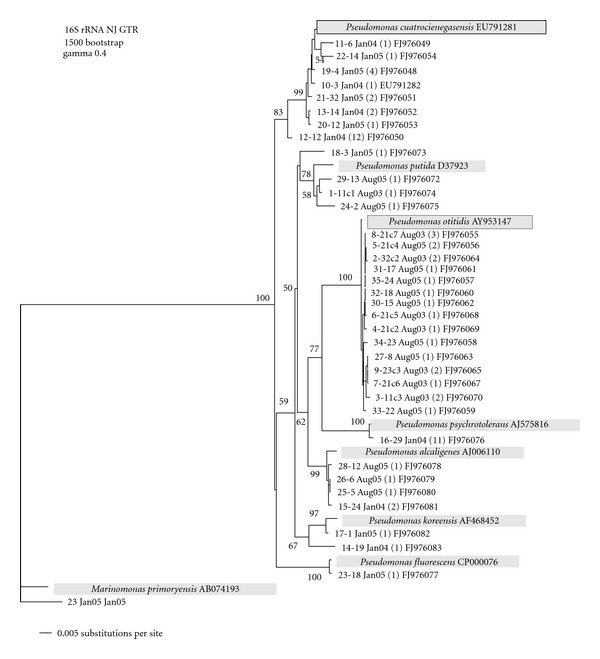
Neighbor-joining tree (GTR, gamma correction 0.40) of 16S rDNA sequences. Sequences are from *Pseudomonas* isolates from Laguna Grande water samples taken in August 2003, January 2004, January 2005, and August 2005. The number of strains having identical fingerprint pattern is in parentheses. The numbers at the nodes are bootstrap values based on 1500 resamplings.

Phylogenetic diversity was defined by the identification of species or lineages as unique 16S rDNA sequences. To determine the seasonal structure of lineages, the 16S rDNA was sequenced from all the unique genotypes as identified by fingerprinting. The Neighbor-joining genealogy of 16S rDNA sequences represents an estimate of the phylogenetic relationship of the 35 genotypes identified by BOX-PCR and is shown in Figure 3. Using a 97% sequence similarity cutoff for the 16S rDNA sequences, the data revealed two numerically dominant clusters. The first cluster (8 sequences representing 24 strains of the total sample) is closely related to *P. cuatrocienegasensis* [5] and was isolated exclusively in winter samples (January 2004 and January 2005), while the second cluster (15 sequences representing 21 strains of the total sample) is closely related to *P. otitidis* and was isolated exclusively in summer samples (August 2003 and August 2005). The seasonal reappearance of phylotypes, identified by 16S rDNA sequences, was not observed at the BOX-PCR fingerprinting level, since all the patterns were different from one sample occasion to the other (Figure 2). These results show that there is seasonal reoccurrence of specific lineages in this site, but the populations that define them have different genotypic composition from one year to the next.

**Figure 4:**
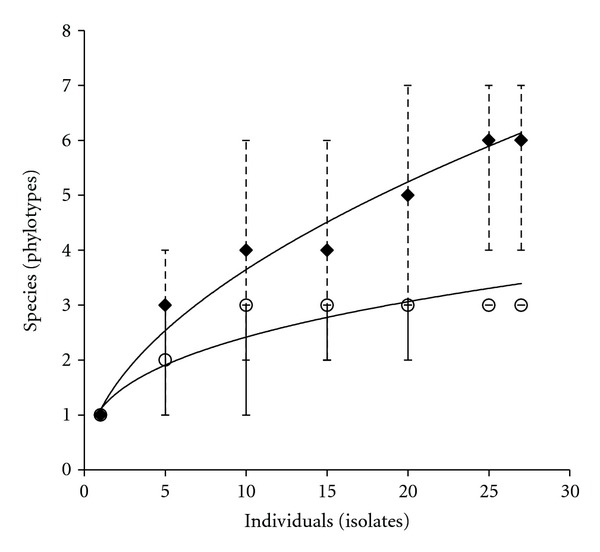
Rarefaction curve constructed with the abundances of the different phylotypes (16S rDNA) in winter and summer samples. Error bars represent the confidence limits for the distributions. ♦ Winter samples (January 2004 and 2005); O summer samples (August 2003 and 2005).

We also analyzed the possibility that the two distinct populations (summer and winter) were not statistically different in terms of the observed diversity, by correcting for sampling size using rarefaction curves. The resulting curves show sampling saturation and that the two populations truly differ in diversity levels (Figure 4). In accordance with rarefaction results, Chao1 richness indices show that the observed number of lineages will not change significantly with more sampling (Table 1).

Additionally, we performed a generalized Fisher's test and a *G* test of independence. Fisher's test was done to evaluate the statistical significance of a seasonal effect on the distribution of phylotypes, as based upon a contingency table. We observed a strongly statistically significant result (*P* = 0.0004998), indicating that the probability of observing the particular arrangement of lineages/seasons by chance is extremely small. The *G* test was done to evaluate the association of phylotypes to sampling seasons and indicated that the probability of finding a particular phylotype is highly dependent on the season (*G* = 108.92; df = 24; *P* = 8.6 × 10^−13^). These results indicate that the observed seasonal distribution of lineages is statistically significant and is not likely due to random events.

**Figure 5:**
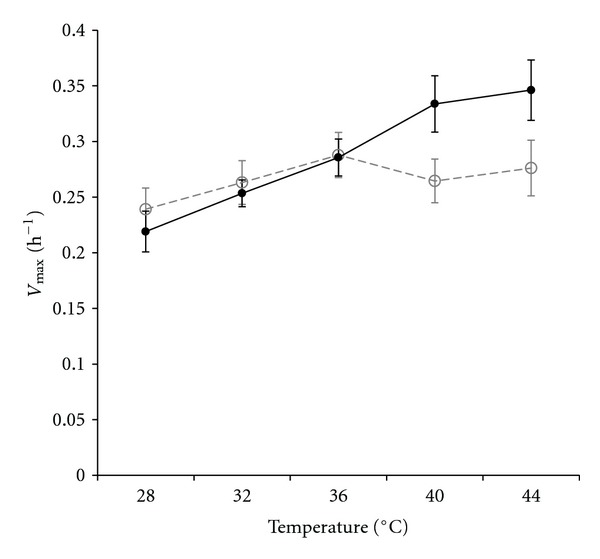
Maximum growth rate, *V*_max⁡_, of a subset of summer (solid line) and winter (dashed line) populations as a function of temperature. Error bars represent 95% confidence intervals, based on 3-fold replication and number of genotypes (9 summer, 6 winter).

Finally, we explored the possibility that *P. cuatrocienegasenesis* and *P. otitidis* populations may differ in their maximum growth rates at different temperatures that can be experienced during summer time (28, 32, 36, 40, and 44°C). We followed the same approach as [36]. We observed that, on average, differences between populations are statistically significant only at 40°C, where *P. otitidis* “summer lineage” grows faster than *P. cuatrocienegasensis* “winter lineage” (Figure 5).

## 4. Discussion

In CCB there is an extraordinary microbial biodiversity, and each site seems to be unique [5–7, 14, 37–39]. As in other places, even if the diversity is high, most of it remains unreachable by traditional culture approaches. Some culturable groups such as *Pseudomonas*, *Bacillus, Exiguobacterium*, and other Firmicutes [6, 13] are an exception. We have found these groups being in high numbers in clone libraries and metagenomes from environmental samples [38, 39] and also have been able to culture them in the laboratory. The microbial diversity information from CCB comes mainly from the study of water systems and ponds, most of which are subject to seasonal fluctuations [1], and nothing is known of the biodiversity changes that occur associated with these environmental cycles. The present study is part of this exploration focusing on the genus *Pseudomonas *and seasonality.

### 4.1. Genetic Structure of Populations (Genotypic Diversity)

BOX-PCR fingerprint analysis and 16S rDNA sequences of all the unique BOX-PCR genotypes were used to determine the temporal structure of the sampled populations. Our results revealed that half of the total number of genotypes were unique (*G*/*N* = 0.5). This diversity value is relatively low in comparison with reported values for *Escherichia coli* (*G*/*N* = 0.73; [40]). However, undersampling, shown by rarefaction curves (data not shown), calls for caution in the interpretation of diversity calculations atat the genotype level.

### 4.2. Seasonal Changes (*P. otitidis* and *P. cuatrocienegasensis*)

Characterization of the phylogenetic diversity leads to the finding of seasonal structure of two numerically dominant lineages: *P. cuatrocienegasensis *and *P. otitidis*. Although diversity may be underestimated at the genotype level due to reduced sample size, we were able to test statistically the correlation between genetic structure and seasonality with a *G* test of independence, a generalized Fisher test, through sampling size correction via rarefaction curves analysis, and by the estimation of the expected richness with nonparametric richness estimator Chao1. *G* test of independence and generalized Fisher test indicate that phylotype (species or lineage) identity is not independent of sampling season (*G* = 108.92; df = 24; *P* = 8.6 × 10^−13^) and that probability of observing the particular arrangement of lineages/seasons by chance is extremely small (*P* = 0.0004998). Rarefaction curves of [41] winter and summer populations showed differentiation between the two and a saturation of diversity for summer samples, giving evidence that both populations differ significantly in their diversity levels (Figure 4). Finally, expected richness indices (Chao1) do not deviate significantly from the observed number of lineages (Table 1). Altogether, these tests indicate that in fact winter and summer populations are statistically different both in their composition and in their diversity levels.

Other studies have found similar patterns in leaf-associated fluorescent pseudomonad populations [41]. Using restriction fragment length polymorphism (RFLP) of leaves samples taken monthly over 3-year period, they found seasonal reappearance of long-term survival ribotypes [41]. In our study, although we were able to discern a seasonal pattern on lineage composition, the factors causing this pattern are more difficult to determine unambiguously. One obvious factor that can be involved in the maintenance of different populations across seasons is temperature. As an attempt to examine this hypothesis, we measured maximum growth rates of the most abundant lineages (*P. otitidis* and *P. cuatrocienegasensis*) at different temperatures. As expected, *P. otitidis* grew faster than *P. cuatrocienegasensis* at high temperatures, but this differential growth was only statistically significant at 40°C. This result provides a clue that temperature can be a relevant environmental factor affecting growth and persistence of isolates, The presented growth rate experiments are far from definitive and must be interpreted with caution, as laboratory conditions invariably differ from the environment in multiple ways beyond that being investigated [42], besides the fact that other environmental parameters that can be associated with temperature changes need to be investigated as well. Nonetheless, these experiments give a good perspective of what can be further done to investigate the factors involved in the observed genetic structure associated with seasonality. We consider that detailed investigation of the physiological responses over a wider temperature range, using more lineages and do measurements with competing isolates, is needed to advance knowledge into the causes of the observed genetic structure of the studied populations.

Another potential explanation for the observed seasonal pattern can be found in the documented transition of certain bacteria into a dormancy state triggered by unfavourable environmental conditions such as oxygen and temperature stress or resource limitation. A recent study by Jones and Lennon [43] demonstrates that only some taxa of the total bacterial community in various lakes were in an active state, and the rest were in a dormant state triggered by environmental stress. Although members of the genus *Pseudomonas *do not form spores, they could enter reversible states of reduced metabolic activity described as viable but nonculturable (VBNC) [44]. Thus, a dormancy/VBNC state could explain the observed seasonal pattern, without excluding other ecological mechanisms (i.e., adaptation). This possibility is one of the limitations that culture-dependent-techniques can have when characterizing microbial diversity. However, several culture-independent techniques have found similar patterns [45] suggesting that the seasonal shifts and reoccurrences of bacterial populations or microbial functional groups occur in the bacterial aquatic communities and, therefore, are not an artefact of the culture-dependent techniques or microbiological procedures [46, 47]. Research in CCB aquatic habitats, including other culturable and nonculturable groups, has recently been published [6] or soon to be [12, 38] that will contribute to determine the generality of the observations here presented.

While we found that lineages or phylotypes (16S rDNA sequences) are seasonally recurrent, genotypes (isolate fingerprints) within each lineage are not, leading to a different genotype composition each year. Despite that undersampling was verified at the genotype level (rarefaction), correcting for sample size at the lineage level, it still gave evidence of differences between summer and winter samples (Figure 4; Table 1). Looking at seasonality on phylotype composition and taking cautiously genotypic composition (fingerprints), we see three possible explanations for our observations: (1) selection associated with seasonality, (2) neutral or stochastic fixation of different genotypes or lineages each season, and (3) artefact due to limited sample size at each sampling date. Given the strong association of phylotypes to sampling season, the selection-mediated possibility is favoured over a purely stochastic explanation. The fact that we do not recover identical fingerprint patterns is debatable due to undersampling and cannot be interpreted as evidence of selective sweeps [47], or simple rapid diversification of bacteria after each seasonal change unless more isolates are analyzed. The third possible explanation relates to the second and implies that genotypes previously “unseen” are present in low numbers; however, this will not necessarily contradict the possibility of seasonal selection acting as an ecological process occurring.

## 5. Conclusion

We showed that the simultaneous utilization of phylogenetic markers and genomic fingerprinting can be used to characterize diversity changes across seasons, and to formulate hypotheses about the potential mechanisms that structure populations. Future experiments that include more phylogenetic groups, larger samples, over extended periods of time, and in controlled laboratory conditions will be necessary to test these hypotheses and further investigate the role of seasonality in the maintenance of lineage (or species) diversity and bacterial diversification in CCB.

The results presented here are the first temporal characterization of the biological composition and dynamics of microorganisms at the CCB study site. The strong correlation of seasonality with the lineage composition contributes with information to formulate future experiments that test hypothesis on the mechanisms involved in the origins and maintenance of microbial diversity in the area.

## Figures and Tables

**Table 1:** Diversity estimates of culturable *Pseudomonas* populations. Total and sample occasion diversity are indicated. Observed diversity in terms of total number of different lineages is indicated as *S*_obs_ and nonparametric richness estimator of the actual number of lineages is indicated as *S*_Chao1_.

Sample occasion	Number of isolates	*S*_obs_	*S*_Chao1_ (SD)
Summer	**27**	**2**	2 (0)
August 2003	15	1	1 (0)
August 2005	12	2	2 (0.05)
Winter	**43**	**8**	9.33 (0.92)
January 2004	31	5	7 (3.74)
January 2005	12	6	9.6 (7.19)

Total	**70**	**8**	10 (3.74)

## References

[1] Minckley W (1969). *Environments of the Bolson of Cuatro Cienegas, Coahuila, Mexico*.

[2] Elser JJ, Schampel JH, Garcia-Pichel F (2005). Effects of phosphorus enrichment and grazing snails on modern stromatolitic microbial communities. *Freshwater Biology*.

[3] Contreras-Arquieta A (1998). New records of the snail Melanoides tuberculata (Muller, 1774) (Gastropoda: Thiaridae) in the Cuatro Cienegas Basin, and its distribution in the state of Coahuila, Mexico. *Southwestern Naturalist*.

[4] Souza V, Espinosa-Asuar L, Escalante AE (2006). An endangered oasis of aquatic microbial biodiversity in the Chihuahuan desert. *Proceedings of the National Academy of Sciences of the United States of America*.

[5] Escalante AE, Caballero-Mellado J, Martínez-Aguilar L (2009). *Pseudomonas cuatrocienegasensis* sp. nov., isolated from an evaporating lagoon in the Cuatro Ciénegas valley in Coahuila, Mexico. *International Journal of Systematic and Evolutionary Microbiology*.

[6] Cerritos R, Eguiarte LE, Avitia M (2011). Diversity of culturable thermo-resistant aquatic bacteria along an environmental gradient in Cuatro Ciénegas, Coahuila, México. *Antonie van Leeuwenhoek, International Journal of General and Molecular Microbiology*.

[7] Bonilla-Rosso G, Peimbert M, Olmedo G (2012). Microbial mat metagenomes reveal common patterns in microbialite community structure and composition. *Astrobiology*.

[8] Escalante AE, Eguiarte LE, Espinosa-Asuar L, Forney LJ, Noguez AM, Souza Saldivar V (2008). Diversity of aquatic prokaryotic communities in the Cuatro Cienegas basin. *FEMS Microbiology Ecology*.

[9] Toribio J, Escalante AE, Caballero-Mellado J (2011). Characterization of a novel biosurfactant producing *Pseudomonas koreensis* lineage that is endemic to Cuatro Ciénegas Basin. *Systematic and Applied Microbiology*.

[10] Moreno-Letelier A, Olmedo G, Eguiarte LE, Souza V (2012). Divergence and phylogeny of Firmicutes from the Cuatro Cienegas Basin, Mexico: a window to an ancient ocean. *Astrobiology*.

[11] Olson DM, Dinerstein E (2002). The global 200: priority ecoregions for global conservation. *Annals of the Missouri Botanical Garden*.

[12] Rebollar EA, Avitia M, Eguiarte LE (2012). Water-sediment niche differentiation in ancient marine lineages of *Exiguobacterium* endemic to the Cuatro Cienegas Basin. *Environmental Microbiology*.

[13] Souza V, Eguiarte LE, Siefert J, Elser JJ (2008). Microbial endemism: does phosphorus limitation enhance speciation?. *Nature Reviews Microbiology*.

[14] Souza V, Siefert J, Escalante AE (2012). The Cuatro Cienegas Basin in Coahuila, Mexico: and astrobiological Precambrian park. *Astrobiology*.

[15] Chesson P (2000). Mechanisms of maintenance of species diversity. *Annual Review of Ecology and Systematics*.

[16] Carson E, Tobler M, Minckley W (2012). Relationships between spatio-temporal environmental and genetic variation reveal and important influence of exogenous selection in a pupfish hybrid zone. *Molecular Ecology*.

[17] Carson E (2005). *Hybridization between Cyprinodon atrorus and C. bifasciatus: history, patterns and dynamics [Ph.D. thesis]*.

[18] Kielwein G (1971). Die isolierung und differenzierung von Pseudomonaden aus lebensmittel. *Archiv für Lebensmittelhygiene*.

[19] Versalovic J, Koeuth T, Lupski JR (1991). Distribution of repetitive DNA sequences in eubacteria and application to fingerprinting of bacterial genomes. *Nucleic Acids Research*.

[20] Häne BG, Jäger K, Drexler HG (1993). The Pearson product-moment correlation coefficient is better suited for identification of DNA fingerprint profiles than band matching algorithms. *Electrophoresis*.

[21] Rademaker J, de Brujin F, Caetano-Anollés G, Gresshoff P (1997). Characterization and classification of microbes byr rep-PCR genomic fingerprinting and computer assited pattern analysis. *DNA Markers: Protocols, Applications and Overviews*.

[22] Oda Y, Wanders W, Huisman LA, Meijer WG, Gottschal JC, Forney LJ (2002). Genotypic and phenotypic diversity within species of purple nonsulfur bacteria isolated from aquatic sediments. *Applied and Environmental Microbiology*.

[23] Lane DJ, Stackebrandt E, Goodfellow M (1991). 16S/23S rDNA sequencing. *Nucleic Acid Techniques*.

[24] Sacchi CT, Whitney AM, Mayer LW (2002). Sequencing of 16S rRNA gene: a rapid tool for identification of *Bacillus anthracis*. *Emerging Infectious Diseases*.

[25] Gordon D, Abajian C, Green P (1998). Consed: a graphical tool for sequence finishing. *Genome Research*.

[26] Thompson JD, Higgins DG, Gibson TJ (1994). CLUSTAL W: improving the sensitivity of progressive multiple sequence alignment through sequence weighting, position-specific gap penalties and weight matrix choice. *Nucleic Acids Research*.

[27] Keane TM, Creevey CJ, Pentony MM, Naughton TJ, McInerney JO (2006). Assessment of methods for amino acid matrix selection and their use on empirical data shows that ad hoc assumptions for choice of matrix are not justified. *BMC Evolutionary Biology*.

[28] Swofford D (2000). *PAUP: Phylogenetic Analysis Using Parsimony*.

[29] Atlas RM, Bartha R (1993). *Microbial Ecology: Fundamentals and Applications*.

[30] Gotelli NJ, Entsminger GL EcoSim 7. 72. http://www.uvm.edu/~ngotelli/EcoSim/EcoSim.html.

[31] Gotelli NJ, Colwell RK (2001). Quantifying biodiversity: Procedures and pitfalls in the measurement and comparison of species richness. *Ecology Letters*.

[32] Chao A (1984). Non parametric estimation of the number of classes in a population. *Scandinavian Journal of Statistics*.

[33] Colwell RK EstimateS: statistical estimation of species richness and shares species from samples. http://purl.oclc.org/estimates.

[34] Sokal R, Rohlf F (1995). *Biometry: The Principles and Practice of Statistics in Biological Research*.

[35] Cooper VS, Bennett AF, Lenski RE (2001). Evolution of thermal dependence of growth rate of *Escherichia coli* populations during 20,000 generations in a constant environment. *Evolution*.

[36] Desnues C, Rodriguez-Brito B, Rayhawk S (2008). Biodiversity and biogeography of phages in modern stromatolites and thrombolites. *Nature*.

[37] Peimbert M, Bonilla-Rosso G, Olmedo G (2012). Comparative metagenomics of microbialites of Cuatro Cienegas: a window to Precambrianfunction. *Astrobiology*.

[38] López-Lozano NE, Bonilla-Rosso G, García-Oliva F (2012). Bacterial communities and nitrogen cycle in the gypsum soil in Cuatro Cienegas Basin, Coahuila. *Astrobiology*.

[39] Borges LGDA, Dalla Vechia V, Corção G (2003). Characterisation and genetic diversity via REP-PCR of *Escherichia coli* isolates from polluted waters in southern Brazil. *FEMS Microbiology Ecology*.

[40] Ellis RJ, Thompson IP, Bailey MJ (1999). Temporal fluctuations in the pseudomonad population associated with sugar beet leaves. *FEMS Microbiology Ecology*.

[41] Jessup CM, Kassen R, Forde SE (2004). Big questions, small worlds: microbial model systems in ecology. *Trends in Ecology and Evolution*.

[42] Jones SE, Lennon JT (2010). Dormancy contributes to the maintenance of microbial diversity. *Proceedings of the National Academy of Sciences of the United States of America*.

[43] Xu HS, Roberts N, Singleton FL (1982). Survival and viability of nonculturable *Escherichia coli* and *Vibrio cholerae* in the estuarine and marine environment. *Microbial Ecology*.

[44] Hullar MAJ, Kaplan LA, Stahl DA (2006). Recurring seasonal dynamics of microbial communities in stream habitats. *Applied and Environmental Microbiology*.

[45] Sutton SD, Findlay RH (2003). Sedimentary microbial community dynamics in a regulated stream: East Fork of the Little Miami River, Ohio. *Environmental Microbiology*.

[46] Fuhrman JA, Hewson I, Schwalbach MS, Steele JA, Brown MV, Naeem S (2006). Annually reoccurring bacterial communities are predictable from ocean conditions. *Proceedings of the National Academy of Sciences of the United States of America*.

[47] de Visser JAGM, Rozen DE (2006). Clonal interference and the periodic selection of new beneficial mutations in *Escherichia coli*. *Genetics*.

